# Stress-related and reproductive hormones in hair from three north Pacific otariid species: Steller sea lions, California sea lions and northern fur seals

**DOI:** 10.1093/conphys/coaa069

**Published:** 2020-09-08

**Authors:** Mandy J Keogh, Angela Gastaldi, Patrick Charapata, Sharon Melin, Brian S Fadely

**Affiliations:** 1 Division of Wildlife Conservation, Alaska Department of Fish and Game, P.O. Box 110024 Douglas, AK 99811-0024, USA; 2 Division of Wildlife Conservation, Alaska Department of Fish and Game, 1300 College Road, Fairbanks, AK 99701, USA; 3 Department of Biology, Baylor University, One Bear Place, Waco, TX 67679, USA; 4 Marine Mammal Laboratory, Alaska Fisheries Science Center, National Marine Fisheries Service, National Oceanic and Atmospheric Administration, 7600 Sand Point Way NE, Seattle, WA 98115

**Keywords:** stress, cortisol, aldosterone, corticosterone, testosterone, hair

## Abstract

Assessing the physiological impact of stressors in pinnipeds is logistically challenging, and many hormones are altered by capture and handling, limiting the utility of metabolically active tissues. Hair is increasingly being used to investigate stress-related and reproductive hormones in wildlife populations due to less-invasive collection methods, being metabolically inert once grown and containing multiple biomarkers of ecological interest. We validated enzyme immunoassays for measuring aldosterone, cortisol, corticosterone, and testosterone in lanugo (natal hair grown *in utero*) samples collected from Steller sea lions (*Eumetopias jubatus*), California sea lions (*Zalophus californianus*), and northern fur seals (*Callorhinus ursinus*). We applied laboratory validation methods including recovery of added mass, parallelism and dilution linearity. We found no effects due to differences in alcohol- versus detergent-based cleaning methods. Further, there were no significant differences in hormone concentrations in hair samples collected immediately after the molt and the subsequent samples collected over 1 year, indicating steroid hormones are stable once deposited into pinniped hair. We found no sex differences in any hormone concentrations, likely due to the lanugo being grown *in utero* and influenced by maternal hormone concentrations. For Steller sea lion and California sea lion pups, we found hormone concentrations significantly differed between rookeries, which warrants future research. Hair provides a novel tissue to explore the intrinsic or extrinsic drivers behind hormone measurements in otariids, which can be paired with multiple health-related metrics to further investigate possible drivers of physiological stress.

## Introduction

Assessing the impact of potential stressors in pinnipeds is logistically challenging and often requires capture and handling (DeRango *et al.*, 2018, Keogh *et al.*, 2013). Many stress-related hormones can be altered during this process, thus limiting the utility of metabolically active tissues, such as blood (Atkinson *et al.*, 2015). The hypothalamic–pituitary–adrenal (HPA) axis activates in response to a stressor leading to the secretion of glucocorticoids (GCs) and a suite of behavioural and physiological changes (Atkinson *et al.*, 2015, Sapolsky *et al.*, 2000, Wingfield *et al.*, 2003, Wingfield, 2005). Further in pinnipeds, aldosterone is influenced by the HPA axis in response to acute stressors (DeRango *et al.*, 2018, Gulland *et al.*, 1999, Keogh *et al.*, 2015). In fact, following an adrenocorticotropic hormone (ACTH) injection, serum aldosterone concentrations increased more rapidly and showed a greater fold increase compared circulating cortisol concentration (Keogh and Atkinson, 2015). Similarly, capture stress induced an increase in circulating aldosterone, cortisol, and corticosterone concentrations in adult Guadalupe fur seals (*Arctocephalus philippii townsendi*) (DeRango *et al.*, 2018). Thus, circulating stress-related hormones measured in blood are influenced by many factors that may be outside the aims of a study and only provide a snapshot of the physiology of an animal. Further, the HPA axis activity and the response to stressors can be influenced by life history traits, environmental conditions and disease processes (Crespi *et al.*, 2013, Dantzer *et al.*, 2014, Dantzer *et al.*, 2013, DeRango *et al.*, 2019, Ensminger *et al.*, 2014). For example, adult female California sea lions (*Zalophus californianus*) experiencing domoic acid (DA) toxicosis had significantly lower serum cortisol concentrations following an ACTH stimulation test suggesting DA exposure is associated with a suppressed adrenal gland response to acute stress in sea lions (Gulland *et al.*, 2012), which may influence the *in utero* environment during gestation.

Hair is increasingly being used to investigate a variety of health-related parameters in wildlife and has the added benefit of being easily collected and stored in remote field settings. GCs are traditionally evaluated when assessing stressors and, in most mammals, cortisol is the primary GC with corticosterone being dominant in rodents (Wasser *et al.*, 2010). The dominant GC is determined by the cortisol: corticosterone (F:B) ratio, where a ratio >1.0 indicates cortisol is the dominant GC. Hair cortisol concentrations have been used as indicators of stress in free-ranging wildlife (Bechshøft *et al.*, 2011, Bryan *et al.*, 2013, Bryan *et al.*, 2015, Macbeth *et al.*, 2012). In polar bears (*Ursus maritimus*), hair cortisol concentrations were influenced by large scale climatic changes associated with the North Atlantic Oscillation index (Bechshøft *et al.*, 2012) and by fatness, whilst hair cortisol and mercury concentrations were related only in males (Bechshøft *et al.*, 2015). In brown bears (*Ursus arctos*), elevated hair cortisol concentrations were associated with reduced natural food sources (e.g. salmon, Bryan *et al.*, 2013), indicating nutritional stress. Further, hair testosterone concentrations can indicate social stress. For example, male and female brown bears in areas of higher population densities near salmon streams had greater hair testosterone concentrations (Bryan *et al.*, 2013). Whilst there has been increasing use of hair to measure hormones in terrestrial mammals, there has been only one study in a pinniped species (Meise *et al.*, 2016). In Antarctic fur seals (*Arctocephalus gazella*), cortisol and testosterone concentrations in hair were influenced by colony density. Further, testosterone concentrations in lanugo (natal hair grown *in utero*) correlated with maternal hair cortisol concentrations, supporting the use of pup hair to assess *in utero* conditions during development in otariids (Meise *et al.*, 2016).

Lanugo is grown during late gestation (~3 months; Rea *et al.,* 2013) and retained until the first molt, which occurs ~4–5 months postbirth in Steller sea lions (*Eumetopias jubatus*) and California sea lions (Braham *et al.*, 1980) and 2 months postbirth in northern fur seals (*Callorhinus ursinus*) (Boltnev *et al.*, 1998, Scheffer, 1961). Hair samples collected prior to the first molt allow for the retrospective assessment of potential stressors during gestation. We chose three adrenal hormones (aldosterone, cortisol, and corticosterone) previously shown to increase in response to stressors in pinnipeds. We also measured testosterone concentrations; a steroid hormone influenced by social stressors. The objectives of this study were to (i) validate enzyme immunoassays (EIA) and measure steroid hormones from lanugo in three otariid species, (ii) compare detergent and alcohol-based cleaning methods, (iii) investigate the stability of steroid hormones after deposition in hair, and (iv) investigate the variability in hormone concentrations in lanugo, including sex and rookery differences within species and *in utero* DA exposure in California sea lions.

## Materials and methods

### Sample collection

We collected lanugo samples from Steller sea lions, California sea lions, and northern fur seals (Table 1). For the sea lion samples, hair was shaved from a patch lateral to the spine and dorsal to the rear flipper insertion, whereas samples from the northern fur seals were collected with surgical scissors, resulting in smaller samples. We did not attempt to separate guard hair and undercoat in the fur seal samples. Further, northern fur seals rely on hair as their insulation as opposed to blubber in sea lions (Liwanag *et al.*, 2012), a second reason to limit sample collection. Given the limited sample size from northern fur seal pups, additional samples from subsistence hunted subadult males collected by the Aleut Community of St. Paul Island were used for validation of the EIAs. Once the methods were finalized, we measured four steroid hormones in samples from 50 live Steller sea lion pups, 46 dead California sea lion pups and 15 live northern fur seal pups (Table 1). An additional 12 California sea lion pups were sampled post-mortem whose dams (*n* = 7) were diagnosed with suspect DA toxicosis and were euthanized. All hair samples were stored in either polypropylene bags or 50-ml conical tubes at ambient temperature if dry or frozen if wet until processed for analysis. Body mass of Steller sea lion pups was measured to the nearest tenth of a kilogram using a hanging electronic scale.

**Table 1:** Sample information including year, location, sex and comparisons made for each species

	Steller sea lion		California sea lion			Northern fur seal	
Sites	Agattu Island, AK	Ulak Island, AK	San Miguel Island, CA	San Nicolas Island, CA	The Marine Mammal Center	St. Paul Island, AK	San Miguel Island, CA
Year	2017		2018		2019	2016 (*n* = 15) 2017 (*n* = 8)	Sept 2018
Sex	18 female 7 male	12 female 13 male	17 female 16 male	7 female 6 male	6 female 6 male	23 male	5 female 10 male
Age class	pup		pup			Subadult	pup
Comparison	sex, rookery, mass		sex, rookery		domoic acid	age class	age class, sex

### Comparing cleaning methods

In order to use archived Steller sea lion hair samples previously washed with 1% Triton™ X-100, we compared two cleaning protocols: a methanol-based method (Cattet *et al.*, 2017, Macbeth *et al.*, 2012) and a detergent-based method (Castellini *et al.*, 2012, Rea *et al.*, 2013). Steller sea lion hair samples (*n* = 50) collected between 2002 and 2005 were divided into two subsamples. One set was washed with 1% Triton™ X-100 for 15 min, followed by multiple rinses with ultrapure water, frozen and then freeze-dried as described by Castellini *et al.* (2012). The second set of samples was washed three times with enough 100% methanol to cover the sample. Hair samples were agitated, and methanol changed with each wash. All hair samples were dampened with 100% methanol and cut with scissors, allowed to air dry and stored in glass scintillation vials. Hormones were extracted from each sample set as described below and cortisol and testosterone were measured to test for statistical differences between the cleaning methods. Since no difference was found between cleaning methods (discussed below), subsequent samples were cleaned using the detergent method.

### Extraction and measurement of hormones

We weighed two 20-mg subsamples from each hair sample into polypropylene tubes (Type I, Sarstedt®). Samples were powdered at 30 KHz for 12 min in a Retsch MM 400 Mixer Mill with two 5-mm steel ball bearings (Retsch Inc., Newtown, Pennsylvania, USA). We extracted hormones following methods used for terrestrial mammals (Bryan *et al.*, 2013, Macbeth *et al.*, 2010). Briefly, 1 ml of 100% methanol was added to the powdered sample and rotated slowly on a benchtop tube rotator for 24 h at room temperature. Following rotation, we removed ball bearings and centrifuged all samples at 10 500 g, 10°C for 13 min and transferred the supernatant to a new polypropylene tube (Type I, Sarstedt®). The remaining pellet was rinsed with 0.3 ml of methanol, agitated, centrifuged again, and supernatant was removed and combined with the previously removed supernatant. Methanol extracts were stored at ≤−20°C until assayed. A subsample of the methanol with extracted hormones were removed, transferred to borosilicate glass vials, dried under forced air and reconstituted in assay buffer from EIA kits.

### Stability of hormones in hair

To determine if hormone concentrations in hair change after deposition with exposure to the natural environment (e.g. water, ultraviolet light, temperature), we shaved hair samples from adjacent sites over the course of 1 year from four female Steller sea lions (three adults, one pup) housed at the Alaska Sealife Center in Seward, AK (60°N, 149°W). The samples were collected lateral to the spine and dorsal to the rear flipper insertion on the right side. The initial samples from three sea lions were collected in October 2016 shortly after the annual molt whilst the fourth sea lion had the first sample collected in early December 2016. Subsequent samples were collected in January or February with the third and final sample collection occurring in June 2017 prior to the next molt. Sea lions were housed primarily in an outdoor exhibit with exposure to natural light cycles and ambient temperatures with periods in an indoor holding pool. Sample collection occurred in conjunction with veterinary health assessments and other research activities under NMFS Permit No. 18534 and in accordance with the Alaska Sealife Center IACUC Protocol No. R12-3-02. Samples were cleaned with the detergent method, and the hormones were extracted using protocol outlined above.

### Laboratory validations

Pools of extracted hormones for male and female Steller and California sea lion pups, and pools from subadult male northern fur seals were made from multiple samples and used to validate EIA kits from Arbor Assay (Ann Arbor, MI, USA) to measure cortisol (K003), corticosterone (K014), aldosterone (K052), and testosterone (K032). We used standard methods including recovery of added mass, parallelism and dilution linearity for the laboratory validations of EIA kits for each hormone (Hunt *et al.*, 2014, Keogh *et al.*, 2013). Assay validations were initially performed with Steller sea lion samples and then applied to California sea lions, and finally, northern fur seals. We assayed all samples in duplicate per manufacturer’s instructions, and all assays included a full standard curve, non-specific binding wells, ‘zero’ (blank) wells, and two controls. For each hormone, the inter-assay and intra-assay coefficients of variation were both <15%. Assay sensitivities were 4.97 pg/ml for aldosterone, 17.3 pg/ml for cortisol, 18.6 pg/ml for corticosterone, and 9.92 pg/ml for testosterone.

### Statistical analysis

Data were analyzed with Systat 13 (Systat Software, Inc., Point Richmond, CA). Normality of data was assessed with probability plots, and if needed, the data were log-transformed. We used a stepwise general linear model with an iterative process of comparing models resulting in the final model including only variables and interactions with a *P* ≤ 0.100. For Steller sea lions the full model used sex and rookery as categorical variables and mass as a continuous variable, and all interaction terms. For California sea lions, we included the categorical variables, sex and rookery and the interaction terms. Repeated measures analysis of variance (ANOVA) was used to evaluate differences in hormone concentration over time and paired t-tests were used to compare cleaning methods. Pearson correlations were used to assess the relationships between hormones (aldosterone, cortisol, corticosterone, testosterone). Means ± SD are reported, and results were considered statistically significant if *P* ≤ 0.050. F:B ratios were also calculated.

## Results

### Laboratory validations

**Figure 1:**
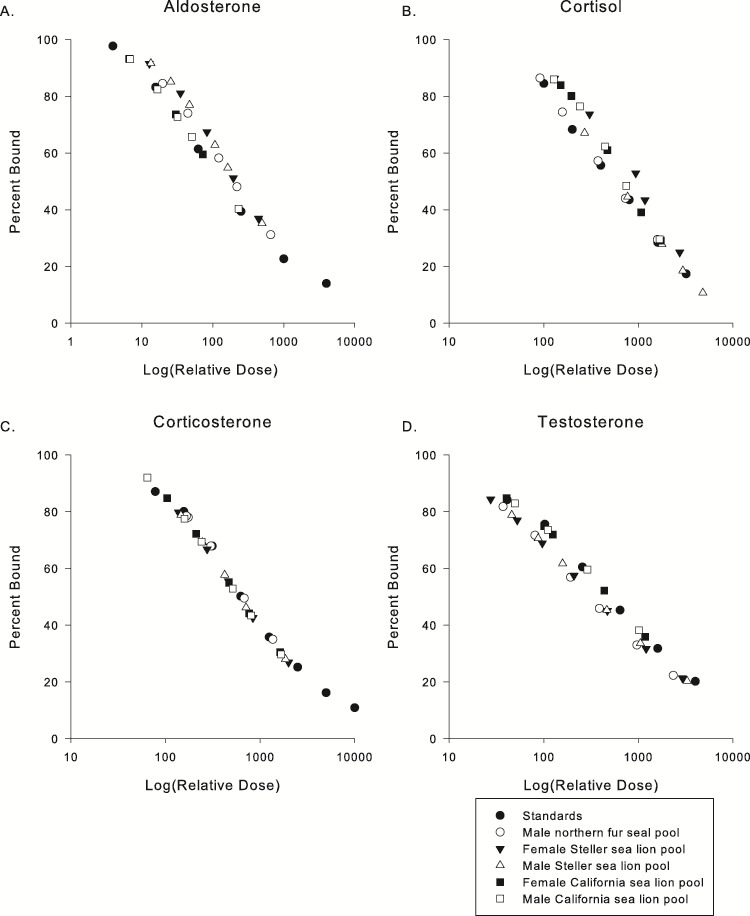
Parallelism and linearity validation results for male and female Steller sea lion and California sea lion pups and male northern fur seal pooled hair extracts for (**A**) aldosterone, (**B**) cortisol, (**C**) corticosterone, and (**D**) testosterone

The slopes of serially diluted methanol extracts, pooled by species and sex, demonstrated linearity and parallelism to the standards (Fig. 1a–d). Further, the slopes for the observed vs expected dose for all EIAs for accuracy tests demonstrated a good fit for the *y*-intercept and the slope of the expected dose alone (Supplemental Table 1).

### Comparing cleaning methods

**Figure 2:**
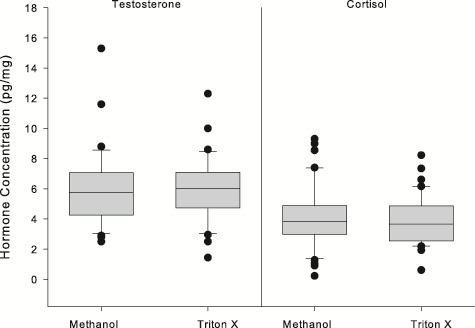
Comparison of the concentration of testosterone (*n* = 32) and cortisol (*n* = 40) in paired samples after being processed using two washing methods: methanol and Triton^TM^ X-100. Box plot: grey box includes data between the 25th and 75th percentile, with a line within each box denoting the median, the whiskers denote the error bars (10 and 90% percentile), and the circles denote outliers

There was no difference in testosterone concentrations (*n* = 32) in samples cleaned with methanol (6.0 ± 2.6 pg/mg) compared to samples washed with detergent (6.1 ± 2.3 pg/mg; paired *t*-test: 0.002, *P* = 0.969) nor was there a difference in cortisol concentrations for samples cleaned with methanol (4.1 ± 2.1 pg/mg) compared to those washed with detergent (3.9 ± 1.7 pg/mg, paired *t*-test: 0.704, *P* = 0.407; Fig. 2).

### Stability of hormones in hair

There was no significant difference in any hormone concentrations in hair samples collected immediately after the molt and the subsequent samples collected from the four Steller sea lions (*P ≥* 0.278; Table 2).

### Steller sea lions

**Figure 3:**
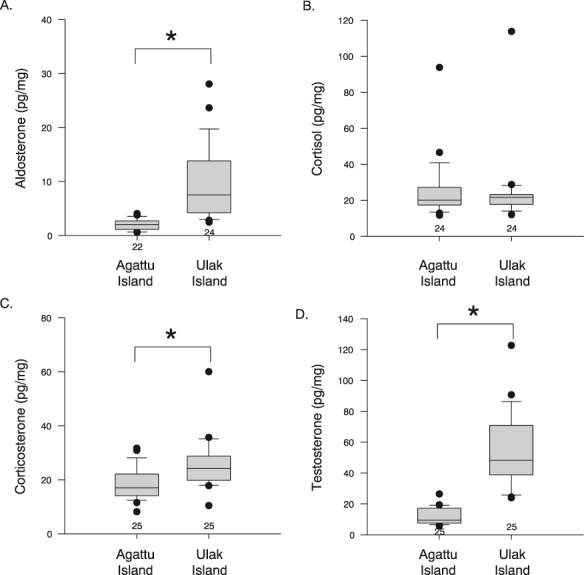
Concentrations of (**A**) aldosterone, (**B**) cortisol, (**C**) corticosterone, and (**D**) testosterone in Steller sea lion pups. There were no differences in hormone concentrations amongst male and female pups. Significant differences between rookeries are denoted by * and sample size noted on graphs. Box plot: grey box includes data between the 25th and 75th percentile, with a line within each box denoting the median, the whiskers denote the error bars (10 and 90% percentile), and the circles denote outliers

Cortisol concentrations ranged between 11.7 and 113.8 pg/mg (24.7 ± 11.7 pg/mg), corticosterone concentrations ranged between 8.2 and 60.0 pg/mg (22.1 ± 8.5 pg/mg), aldosterone concentrations ranged between 0.6 and 28.0 pg/mg (6.2 ± 6.3 pg/mg) and the concentrations of testosterone ranged between 5.8 and 122.7 pg/mg (33.2 ± 27.3 pg/mg). We log transformed the hormone concentrations to meet the assumption of normality before analysis. We found no sex effect in any of the hormones, nor was there a relationship between pup mass and any hormone concentrations. Log aldosterone, log corticosterone and log testosterone concentrations were significantly greater in hair samples from pups on Ulak Island compared to those from Agattu Island (Fig. 3; *P* < 0.001). Whereas, log cortisol concentrations did not differ between sites (Agattu Island 25.3 ± 17.4 pg/mg, Ulak Island 20.7 ± 4.4 pg/mg; *P* = 0.868), even after the one outlier (113.7 pg/mg) was removed (*P* = 0.776). Cortisol concentrations were positively correlated with corticosterone (*r* = 0.588, *P* = 0.001) but not aldosterone or testosterone (*P* = 1.0). A positive correlation was found between corticosterone and testosterone (*r* = 0.534, *P* < 0.001) and between aldosterone and testosterone (*r* = 0.822, *P* = 0.001). The F:B ratio ranged between 0.5 and 3.0 (1.2 ± 0.5); pups on Agattu Island (1.3 ± 0.5;0.6–3.0) had a greater F:B ratio compared to pups sampled on Ulak Island (0.9 ± 0.5; 0.5–1.9; *P* = 0.006).

### California sea lions

**Figure 4:**
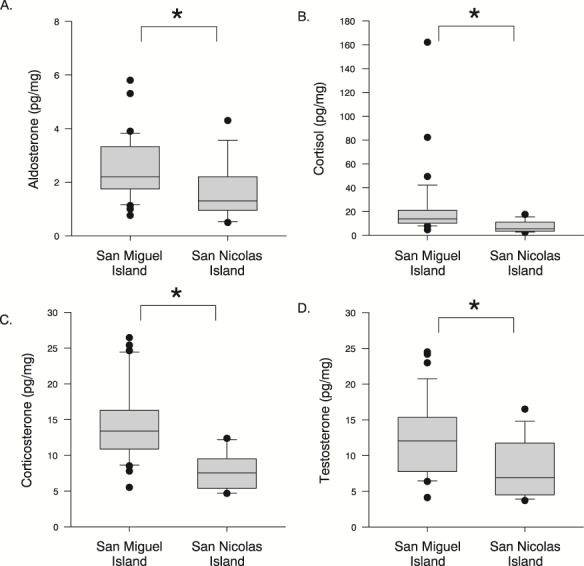
Concentrations of (**A**) aldosterone, (**B**) cortisol, (**C**) corticosterone, and (**D**) testosterone were all significantly higher in California sea lion pups sampled on San Miguel Island (*n* = 33) compared to San Nicolas Island (*n* = 13). There were no differences in hormone concentrations amongst female and male pups. Box plot: grey box includes data between the 25th and 75th percentile, with a line within each box denoting the median, the whiskers denote the error bars (10 and 90% percentile), and the circles denote outliers

Hormone concentrations for California sea lion pups sampled on San Miguel Island (SMI) and San Nicolas Island (SNI) were as follows: cortisol concentrations ranged between 2.6 and 162.1 pg/mg (18.3 ± 25.5 pg/mg), corticosterone concentrations ranged between 4.7 and 26.4 pg/mg (12.5 ± 5.5 pg/mg), aldosterone concentrations ranged between 0.5 and 5.8 pg/mg (2.2 ± 1.2 pg/mg) and the concentrations of testosterone ranged between 3.7 and 24.5 pg/mg (11.0 ± 5.2 pg/mg). We log transformed the cortisol concentrations to meet the assumption of normality before analysis. Hair samples from pups sampled on SMI had higher aldosterone (SMI 2.5 ± 1.1 pg/mg; SNI 1.6 ± 1.0; *P* = 0.015), cortisol (SMI 22.7 ± 28.9 pg/mg; SNI 7.2 ± 4.6 pg/mg; *P* < 0.001), corticosterone (SMI 14.3 ± 5.2 pg/mg; SNI 7.8 ± 2.6 pg/mg; *P* < 0.001), and testosterone (SMI 12.2 ± 5.3 pg/mg; SNI 8.1 ± 4.0 pg/mg; *P* = 0.008) concentrations compared to pups sampled on SNI (Fig. 4). There was no sex difference in any hormone. Cortisol concentrations were correlated with the concentrations of testosterone (*r* = 0.520, *P* = 0.002) and corticosterone (*r* = 0.606, *P* < 0.001). There were no correlations between any other hormones. The F:B ratio for California sea lion pups ranged from 0.25 to 9.75 (1.4 ± 1.5) and the F:B ratio of pups sampled on SMI was more variable and significantly greater (1.6 ± 1.7) than the F:B ratio in SNI pups (0.9 ± 0.5; *P* = 0.034). We log transformed the 12 hair samples from the Marine Mammal Center and compared hormone concentrations between pups from dams with suspect DA toxicosis to offspring from dams with unknown status (UNK). Aldosterone concentrations were greater in DA pups (DA: 16.1 ± 11.1 pg/mg, UNK: 4.8 ± 2.1 pg/mg; *P* = 0.006;) and whilst testosterone concentrations were greater in DA pups (172.1 ± 196.0; 15.0 ± 7.8 pg/mg), the difference was not significant (*P* = 0.059). There was no difference in concentrations of cortisol (DA: 21.3 ± 14.0, UNK: 30.4 ± 23.8 pg/mg; *P* = 0.694) or corticosterone (DA: 45.9 ± 26.0 pg/mg, UNK: 33.1 ± 20.8 pg/mg; *P* = 0.509), nor did the F:B ratio differ (DA 0.5 ± 0.1, UNK 0.8 ± 0.3; *P* = 0.081).

**Table 2:** Hormone concentrations in hair samples collected from captive SSL over the course of 1 year

	Sample 1 *n* = 4	Sample 2 *n* = 4	Sample 3 *n* = 3	
Aldosterone (pg/mg)	1.1 ± 0.5	1.3 ± 0.4	1.3 ± 0.5	F_2,4_ = 0.468, *P* = 0.656
Cortisol (pg/mg)	3.8 ± 1.0	5.8 ± 4.4	4.0 ± 0.9	F_2,4_ = 0.665, *P* = 0.563
Corticosterone (pg/mg)	6.8 ± 1.3	8.4 ± 4.0	8.4 ± 4.3	F_2,4_ = 0.478, *P* = 0.651
Testosterone (pg/mg)	2.6 ± 1.7	2.5 ± 0.8	4.3 ± 2.8	F_2,4_ = 1.794, *P* = 0.278

Samples 1 were collected shortly after the molt, Samples 2 and 3 were collected adjacent to Samples 1 at ~4-month intervals. There was no significant difference between samples using repeated measures ANOVA analysis.

**Figure 5:**
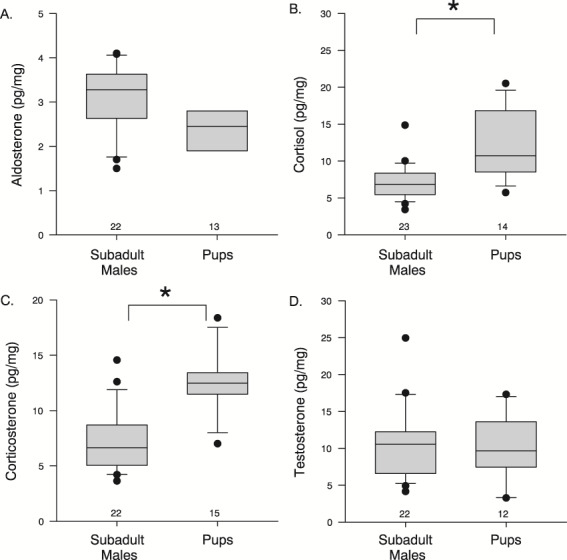
Concentrations of (**A**) aldosterone, (**B**) cortisol, (**C**) corticosterone, and (**D**) testosterone in subadult male northern fur seals and pups. There were no differences in hormone concentrations between male and female pups. * denotes significant differences found between groups and sample size noted on each graph. Box plot: grey box includes data between the 25th and 75th percentile, with a line within each box denoting the median, the whiskers denote the error bars (10 and 90% percentile), and the circles denote outliers

### Northern fur seal

Hormone concentrations in the subadult males ranged between 3.4 and 14.8 pg/mg (7.2 ± 2.4 pg/mg) for cortisol, 3.6 and 14.6 pg/mg (7.2 ± 2.8 pg/mg) for corticosterone, 1.5 and 4.1 pg/mg (3.0 ± 0.8 pg/mg) for aldosterone, and 4.1 and 24.9 pg/mg (10.37 ± 4.8 pg/mg) for testosterone. The small sample size limited the number of hormones analyzed in pups (Table 1). There was no sex effect for any of the hormones in pups (*P* < 0.392). Pup hormone concentrations ranged between 5.7 and 20.5 pg/mg (12.1 ± 4.6 pg/mg) for cortisol, 7.0 and 18.4 pg/mg (12.7 ± 2.9 pg/mg) for corticosterone, 0.9 and 4.1 pg/mg (2.4 ± 0.9 pg/mg) for aldosterone, and 3.3 and 17.3 pg/mg (10.0 ± 4.4 pg/mg) for testosterone. When we compared hormone concentrations between age classes, cortisol (*P* < 0.001) and corticosterone (*P* < 0.001) were higher in pups compared to subadults (Fig. 5). There was no difference between age classes for testosterone (*P* = 0.845) whilst aldosterone was higher but not significantly in subadult males (*P* = 0.069). Cortisol concentrations were positively correlated with corticosterone concentrations (*r* = 0.633, *P* < 0.001), but no other hormones were correlated (*P* ≥ 0.113). The F:B ratio in pups and subadults combined ranged between 0.3 and 2.3 (1.1 ± 0.5).

## Discussion

### Method development

Our findings support the use of otariid pinniped hair samples for measuring steroid hormones providing a stable, less-invasive tissue to collect that can be easily stored. Further, we found no difference in hormone concentrations between two cleaning methods supporting the use of either method. The stability of steroid hormones in hair and whether hormones ‘leach’ out or degrade with exposure to the environment has been questioned (Sheriff *et al.*, 2011). Pinnipeds are amphibious, spending a significant amount of time in seawater with alternating periods on land exposing hair to a wide range of environmental conditions. We found no difference in the concentrations of hormones when hair samples were collected from captive Steller sea lions over the course of 1 year between their annual molt.

### Hormone concentrations in otariid hair

GCs are traditionally evaluated when assessing stressors and, in most mammals, cortisol is the primary GC (F:B ratio > 1.0) with corticosterone being dominant in rodents (Wasser *et al.*, 2010). In California sea lions, the F:B ratio in excised adrenal glands was 1.5, supporting cortisol as the dominant GC in pinniped (DeRoos *et al.*, 1961). Most cortisol dominant mammals have F:B ratio in serum ranging between 7.5 and 49 (Koren *et al.*, 2012). Given the low F:B ratio observed in California sea lions, we calculated the F:B ratio in hair and found a similar mean in the F:B ratio in California sea lion pups, Steller sea lion pups, and northern fur seal pups and male subadults. Interestingly, 40% of California sea lions and northern fur seals and 27% of Steller sea lions had F:B ratio < 1.0. The variability in the GC ratios we found were not completely surprising as a recent study found the F:B ratios in serum ranged between 6.5 and 16.2 in Guadalupe fur seals during an acute stress response (DeRango *et al.*, 2018). We also found cortisol and corticosterone were correlated, explaining between 58 and 63% of the variability in the three otariid species. Similar findings were found across cortisol-dominant taxa with individuals having both higher circulating cortisol and corticosterone concentrations while undergoing an acute stress response due to handling (Koren *et al.*, 2012). Whilst individuals with higher cortisol concentrations had high corticosterone concentrations, nearly half of the variability in GCs in our study was not explained by this relationship, suggesting that measuring both cortisol and corticosterone in otariids may be useful when assessing potential stressors.

We were able to detect aldosterone for the first time in otariid hair. We found aldosterone concentrations greater in Steller sea lion pups and comparable between California sea lion and northern fur seal pups. Our results provide a novel method to measure aldosterone in otariid pinnipeds providing a tool to explore differences in aldosterone concentrations. For example, aldosterone sensitivity to stress may be age dependent, as aldosterone rapidly increases in response to capture stress in adult otariids (DeRango *et al.*, 2018); yet, this sensitivity appears muted in acute stress response in pups compared to cortisol (Keogh *et al.,* 2013, DeRango *et al.,* 2018). Stress-related hormones in hair would allow for comparisons of baseline concentrations between demographic groups independent of an acute stress response.

Testosterone is a reproductive hormone and a potential marker of social stress. We found hair testosterone concentrations comparable between California sea lions and northern fur seals, whilst Steller sea lion pups had higher concentrations. Whilst we did not assess what factors may contribute to these species’ differences, it may be of note that Steller sea lions are the largest otariid. In our study, we found no sex difference in hair testosterone concentrations, likely due to our samples being grown *in utero* and influenced by maternal hormones (Dantzer *et al.,* 2013) as well as all animals being sexually immature. Similarly, no sex difference was found in hair testosterone concentrations in coyote pups (Schell *et al.*, 2017) or Antarctic fur seal pups (Meise *et al.*, 2016). In Antarctic fur seal pups, hair testosterone concentrations were similar between dam and pup with both having higher testosterone concentrations in the denser rookery (Meise *et al.*, 2016). Further, hair testosterone concentrations in the pups were positively correlated with maternal hair cortisol concentrations supporting the stress experienced by the dam during gestation influences the *in utero* environment and the hormones in which the fetus is exposed to during development, potentially playing a role in foetal programming.

### Differences in hormone concentrations by rookeries

We sampled two rookeries each for California and Steller sea lion pups and found significant differences in concentration of hormones between sites. In Steller sea lions, hair cortisol concentrations did not differ between rookeries at either island, whilst aldosterone, corticosterone, and testosterone were all higher in pups from Ulak Island. These findings were somewhat surprising as we expected either no difference or for hormone concentrations to be greater at Agattu Island. Both Agattu and Ulak Islands are within the endangered western distinct population segment, which is further divided into seven metapopulations based on diet and population trends (Fritz *et al.*, 2019, York *et al.*, 1996). The western Aleutian Islands metapopulation includes Agattu Island and had a −6.47% decline in pup production between 2002–2018 whilst the central Aleutian Islands metapopulation, which includes Ulak Island, underwent a smaller decline of −2.08% (Sweeney *et al.*, 2019). Further, both sites are west of the Samalga Pass, with Atka mackerel (*Pleurogrammus monopterygius*) as the dominate prey of females and have fewer and less diverse fish species in their diets compared to females east of Samalga Pass (Sinclair *et al.*, 2013, Sinclair *et al.*, 2005). Given these population trajectories and diet characteristics between our sites, we expected the hair cortisol concentrations would not differ between sites. We may find a difference in hair cortisol concentrations if comparisons were made between other Steller sea lion sites with differing diets or pup production trends, as Myers *et al.* (2010) found serum cortisol concentrations differed between geographic regions of Steller sea lion. The higher concentrations in aldosterone, corticosterone, and testosterone between sites warrants further study, especially given Agattu Island had a greater decline in pup production and lower hormone concentrations compared to Ulak Island. Population trends and diet may not be the only factors influencing the *in utero* exposure of stress-related hormones. Pups from Agattu Island have high mercury concentrations in their lanugo compared to pups sampled at rookeries to the east, though Ulak Island was not sampled (Rea *et al.*, 2013). Mercury is an endocrine disrupter that acts directly and indirectly on the adrenal glands altering the secretion of hormones in seals (Freeman *et al.*, 1974, Sangalang *et al.*, 1976). Future studies measuring adrenal hormones and mercury concentrations in hair samples from Steller sea lions would be able to explore the potential impacts of *in utero* mercury exposure on the endocrine system.

In California sea lion pups, all four hormones were higher in pups sampled on SMI compared to SNI, though the sample size on SNI was small. The colony differences in stress-related hormones could be due to several factors that can be investigated in future studies. Both islands are within the Channel Islands off the Southern California coast and whilst females from these two colonies foraged in different areas, they travelled comparable distances with similar diets (Kuhn *et al.*, 2014, Melin *et al.*, 2008, Peterson *et al.*, 2016). We sampled dead pups at both colonies in 2018, a comparatively good year for pup survival and condition. Rookery differences in serum cortisol concentrations were also found in clinically healthy California sea lion pups sampled at rookeries within the Gulf of California (Padernera-Romano *et al.*, 2010). Besides diet and foraging differences between the colonies, other factors such as disease, parasite or algal blooms and associated DA toxicosis may differ between the colonies and foraging areas and contribute to the differences (Goldstein *et al.*, 2009, Lyons *et al.*, 2016).

DA is a neurotoxin produced by diatoms with increasing episodic events along the California coast driven by oceanographic conditions. In California sea lions, DA exposure is through diet (Lefebvre *et al.*, 1999) and associated with seizures and atrophy of the hippocampus, as well as abortions and premature live births (Brodie *et al.*, 2006, Greig *et al.*, 2007), higher eosinophil counts, and lower serum cortisol concentrations (Gulland *et al.*, 2012). DA toxicosis was associated with significantly lower serum cortisol concentrations following the ACTH stimulation test whilst non-DA exposed females had the expected positive correlation between ACTH and cortisol, suggesting that DA exposure was associated with a suppressed adrenal gland response to acute stress in sea lions (Gulland *et al.*, 2012), which may influence the adrenal hormone concentration in pup lanugo. We measured hormone concentrations in hair grown *in utero* associated with suspected DA in the dam (*n* = 7) compared to solitary pups that stranded without a dam (*n* = 5). Interestingly, suspect DA pups had higher concentrations of aldosterone compared to pups with unknown DA exposure, whereas no difference was found in the GCs. Our sample size was small, and whilst we had suspected cases of DA exposure in the dam, we did not know what, if any, exposure the solitary pups may have had. A larger study is needed to explore the influence of DA exposure *in utero* (Lefebvre *et al.*, 2018) and measuring stress-related hormones in hair samples from California sea lions provides a novel method that may prove useful when assessing endocrine function in the dam or exposure to *in utero* stressors.

## Conclusion

We found that multiple stress-related hormones were measurable in lanugo samples from three North Pacific otariid species: Steller sea lion, California sea lions, and northern fur seals. Steroid hormone concentrations did not significantly change once deposited in hair following the annual molt. Interestingly, we found a difference in the stress-related hormones between rookeries in Steller and California sea lion pups, though we did not test what factors may be driving the differences in hormone concentrations. We also found a difference in some stress-related hormones in California sea lion pups whose dams had suspected DA exposure. Using hair to measure stress-related and reproductive hormones provides a less-invasive method to assess these hormones in wild populations. Measuring hormones in a stable tissue that is also used to measure dietary isotope signatures and mercury concentrations provides a tool to investigate the relationships between these factors as well as other population and individual characteristics in future studies (Fleming *et al.*, 2018).

## Funding

This work was supported by the National Oceanic and Atmospheric Administration [NA16NMF4390029] to the Alaska Department of Fish and Game.

## Supplementary Material

Supplemental_Table_1
