# Supplementary material for: Stress-related and reproductive hormones in hair from three north Pacific otariid species: Steller sea lions, California sea lions and northern fur seals
Source: Conserv Physiol. 2020 Sep 8;8(1):coaa069. doi: 10.1093/conphys/coaa069 (PMC7437363; doi:10.1093/conphys/coaa069)
Supplement: Supplemental_Table_1 [file supplemental_table_1.docx]

Supplemental Table 1. Accuracy validation results for four steroid hormones (aldosterone, cortisol, corticosterone, and testosterone) in pooled hair extracts for male and female Steller sea lion pups, California sea lion pups, and subadult male northern fur seals. We also report the percent recoveries ± standard error.

|  |  | Aldosterone | Cortisol | Corticosterone | Testosterone |
| --- | --- | --- | --- | --- | --- |
| Steller sea lion | Female | 1.2186x - 0.591 R² = 0.799  128.7 ± 12.7% | 0.9933x + 55.554  R² = 0.997  98.6 ± 7.3% | 1.0046x+23.205  R^2^=1.0  102.5 ± 0.7% | 0.9637x-303.92  R^2^=0.988  78.0 ± 7.2% |
|  | Male | 0.9602x + 20.634 R² = 1.0  110.5 ± 7.6% | 0.9318x + 113.48 R² = 0.939  107.1 ± 3.2% | 1.0052x+209.43  r^2^=0.995  102.0 ± 3.3% | 1.0646x-333.84  R^2^=0.982  92.1 ± 8.9% |
| California sea lion | Female | 0.8909x + 95.304 R² = 0.987  109.6 ± 11.6% | 0.975x + 108.87 R² = 0.968  108.1 ± 13.3% | 1.1259x + 70.54 R² = 0.998  82.5 ± 2.9% | 1.0171x - 32.765 R² = 0.990  87.6 ± 10.5% |
|  | Male | 1.0901x - 34.536 R² = 0.999  94.7 ± 10.8% | 0.837x + 77.421 R² = 0.950  64.0 ± 8.7% | 0.8582x - 179.98 R² = 0.997  78.9 ± 2.1% | 0.8283x + 139.49 R² = 0.995  100.7 ± 8.5% |
| northern fur seal | Male | 1.1824x + 7.1875 R² = 0.961  147.9 ± 9.7% | 1.0112x +22.483  R^2^=0.945  102.6 ± 9.4% | 1.032x+146.85  R^2^=0.977  111.2 ± 32.3% | 1.191x+88.27  R^2^=0.997  126.8 ± 8.6% |
